# School performance and later diagnoses of nonaffective psychoses, bipolar disorder, and depression

**DOI:** 10.1111/acps.13481

**Published:** 2022-08-07

**Authors:** David Gyllenberg, Tiina Ristikari, Ian Kelleher, Antti Kääriälä, Mika Gissler

**Affiliations:** ^1^ Department of Child Psychiatry and INVEST Research Flagship Center University of Turku and Turku University Hospital Turku Finland; ^2^ Department of Adolescent Psychiatry University of Helsinki and Helsinki University Central Hospital Helsinki Finland; ^3^ Finnish Institute of Health and Welfare Helsinki Finland; ^4^ Itla Children's Foundation Helsinki Finland; ^5^ Department of Psychiatry Royal College of Surgeons in Ireland Dublin Ireland; ^6^ Lucena Clinic Dublin Ireland

**Keywords:** academic performance psychotic disorders, bipolar disorder, depression, epidemiology

## Abstract

**Objective:**

It is unclear whether there are differences between specific school subject performance and later psychiatric disorders. We examined whether mean grade point average (MGPA) and specific school subjects associated with diagnoses of nonaffective psychoses, bipolar disorder and depression.

**Methods:**

In this register‐based study, we studied the Finnish population born in 1987 who had available MGPA and six specific school grades (age = 15.4–16.4 years; *n* = 50,508). Grades were analyzed with smoothing splines. Covariates included sex, urbanicity, parental education level and parental diagnosed psychiatric disorders. Outcomes were incident nonaffective psychosis, bipolar disorder and depression diagnosed in specialized services until year 2015 (age = 28.0–28.9 years).

**Results:**

During the follow‐up, 727 individuals were diagnosed with nonaffective psychoses, 489 with bipolar disorder and 3492 with depression. MGPA was inversely associated with all outcomes. In multivariate models including specific school subjects and covariates, the school subject with largest risk ratios (RR) was Physical Education (RR and Bonferroni‐corrected confidence interval [CI] at −1.5 SD: nonaffective psychoses 1.63, 1.36–1.95; bipolar disorder 1.64, 1.30–2.05; depression 1.72, 1.53–1.93). Higher grades in Art were associated with nonaffective psychoses and depression (RR and Bonferroni‐corrected CI at +1.5 SD: nonaffective psychoses 1.48, 1.11–1.96; depression 1.22, 1.07–1.38).

**Conclusion:**

There was a robust association between poorer scores on Physical Education and risk for psychosis, bipolar disorder and depression. Higher grades in Art were also associated with risk for later disorders. Subject specific school performance may be more informative about mental disorder risk than overall school performance.

**Significant outcomes**

- Mean grade point average school grades were inversely associated with later diagnoses of nonaffective psychoses, bipolar disorder and depression.
- Low grades in Physical Education showed the strongest independent effect for all diagnoses, but high grades in Art was also nonlinearly associated with nonaffective psychoses and depression.

**Limitations**

- No direct measures of cognitive performance from neuropsychological tests were available.
- Data on psychiatric disorders that were not diagnosed in specialized services were lacking.

## 1 INTRODUCTION

Psychosis, bipolar disorder and depression have been shown to associate with premorbid lower neurocognitive functioning and school performance in large cohort studies.^1^, ^2^, ^3^, ^4^, ^5^, ^6^ However, excellent school performance has also been linked to both later bipolar disorder^2^ and nonaffective psychoses among females.^3^ Describing specific types of school performance in cohort data can help elucidate neurocognitive trajectories that precede onset of diseases.

A few studies have analyzed the impact of specific school subjects on later diagnoses. At ages 7–12 years, low grades particularly in sports and handicrafts predicted schizophrenia in adulthood.^4^ This in line with well‐documented premorbid deficits and delays in motor function associating with later psychoses.^7^, ^8^ At age 8 years, being talented in oral presentations and drawing also predicted psychotic‐like symptoms.^9^ At ages 15–16 years, obtaining the lowest grades in several school subjects predicted later schizophrenia,^1^ while the highest grade in various academic and creative subjects predicted bipolar disorder.^2^ Intriguingly, attending creative courses in high school or university has also been shown to predict schizophrenia, bipolar disorder and depression diagnoses.^10^ To our knowledge, no studies to date have examined both high and low grades of several specific school subjects in relation to risk for later psychoses, bipolar disorder and depression.

### 1.1 Aims of the study

We investigated whether school performance predicted treatment for three major psychiatric disorders—nonaffective psychoses, bipolar disorder and depression—in a total birth cohort. Given that both low and high grades have been associated with some of these disorders, we assessed whether nonlinear associations were present. Our first aim was to examine whether mean grade point average aged 15 to 16 years predicted the above diagnostic outcomes. Our second aim was to investigate independent associations between performance on specific school subjects and later diagnoses.

## 2 METHODS

### 2.1 Study design, setting, and participants

We used the nationwide Finnish Birth Cohort (FBC) 1987, which is managed by the Finnish Institute of Health and Welfare in Finland. The cohort contains various linked information from national registers for all live‐births in Finland in 1987.^11^ The school grades and covariates were assessed on May 31, 2003, that is, by the end of the ninth grade of compulsory primary education, assuming that the cohort members had started school the year turning 7 years and had completed primary education on time. Follow‐up lasted from June 1, 2003, when the cohort members were 15.4–16.4 years old depending on their date of birth, to December 31, 2015, at age 28.0–28.9 years. We excluded subjects who had died or emigrated, had been diagnosed with the studied diagnoses, or had missing values for the studied school grades prior to the start of follow‐up.

### 2.2 Data sources

To link register‐based information, we used the personal identification number (PIN) assigned to all Finnish citizens and residents by the Digital and Population Data Services Agency. We used information from the Medical Birth Register to identify subjects born in Finland in 1987, date of birth and sex; the Digital and Population Data Services Agency to identify cohort members' parents, residential location and emigration; the Joint Application Register to retrieve school grades; Statistics Finland to retrieve dates of death and parental education level; and the Care Register for Health Care to retrieve dates and diagnoses of inpatient and outpatient visits in public specialized services.

The Care Register for Health Care is maintained by the Finnish Institute for Health and Welfare, and it includes records on inpatient treatment since 1969 and on public outpatient and emergency room visits since 1998. The records include data on the PIN, the start and end dates of each visit, a primary diagnosis and optional secondary diagnoses. The register has been extensively used in epidemiologic studies,^11^, ^12^ and the diagnostic validity of the register‐based diagnoses of schizophrenia^13^ and type 1 bipolar disorder^14^ has been assessed.

The Joint Application Register was used to retrieve data on school performance in the final (ninth) year of compulsory primary education. The register is maintained by the National Board of Education and includes grade data for subjects who after primary school apply for secondary education through a national application system. The records include the personal identification number, the mean grade point average of compulsory school subjects, grades of specific school subjects, and the time of the application.

### 2.3 Outcomes

The outcomes were time to incident diagnoses of nonaffective psychoses, bipolar disorder and depression in specialized services. Both primary and secondary and outpatient or inpatient diagnoses were taken into account. All diagnostic codes during the follow‐up period were recorded using the International Statistical Classification of Diseases and Related Health Problems, tenth revision (ICD‐10). We defined nonaffective psychoses with ICD‐10 codes F20‐F22, F24, F25 and F28, and we defined bipolar disorder and depression with ICD‐10 codes F30‐F31 and F32‐F33, respectively. Subjects could be recorded with more than one outcome diagnosis.

### 2.4 School performance variables

Finnish school subjects are recorded on a scale from four to ten, with four being a failing grade, five being the lowest passing grade and ten being the highest. The mean grade point average is the arithmetic mean of compulsory academic subjects. The national core curriculum includes uniform evaluation criteria for the schools what kind of knowledge and skills are needed to receive the grade eight (good) at the end of compulsory primary education.^15^ To study specific school subjects, we used school subjects that had a low rate of missingness and encompassed a range of academic and nonacademic subjects: Native language (Finnish or Swedish), Mathematics, Physical Education, Handicrafts, Art and Music. We scaled all grade points to have a mean value of 0 and a standard deviation of 1 to allow for comparison with international studies.

### 2.5 Covariates

We assessed covariates that were potentially associated with both school or cognitive performance and the studied outcomes: sex, parental education level, psychiatric disorders diagnosed before follow‐up, parental history of any psychiatric diagnosis and urbanicity of residency area.^1^, ^2^, ^3^, ^16^, ^17^, ^18^, ^19^, ^20^, ^21^ Maternal and paternal education level were defined as primary, secondary, and tertiary education level depicting the highest education received by year 2003. Parental history of any psychiatric diagnosis were defined as present if either parent had a record of the a diagnosis anytime from the start of the Care Register for Health Care in 1969 to May 31, 2003. The ICD codes for any diagnosis are described in the Supplement. The urbanicity of residency was defined as urban if the subject lived in an area that classifies as urban according to Finland's Environmental Administration,^22^ that is, an agglomeration with more than 15,000 residents; otherwise it was classified as rural.

### 2.6 Statistical analyses

We described the data by extracting the number of subjects at start of follow‐up, person‐years, number of events and cumulative incidence at end of follow‐up for each outcome. We stratified the descriptive analyses by covariate status and by standardized school grades at z‐scores <−1.5, −1.5 to <−0.5, −0.5 to <0.5, 0.5 to <1.5 and ≥1.5. To obtain the cumulative incidence, we calculated the 1—survival rate from Kaplan–Meier analyses. The outcomes were coded as the time from start of follow‐up to the time of the event. We censored subjects at time of the event, emigration (n = 1551), death (n = 234) or end of follow‐up, whichever came first. We conducted full case analyses.

To investigate associations between school grades and later diagnoses, we fit generalized additive models (GAM) with Poisson distribution and with the log of follow‐up time as offset. The time‐to‐event outcomes were defined as in the descriptive analyses. To allow for nonlinear associations, we modeled the standardized school grades as functions of thin plate smoothing splines^23^ that were included in the GAMs. The smoothing parameter describes the level of smoothness or how much the spline bends from completely linear function to a nonlinear function that follows all data points in thin spline. To avoid overfitting but maintaining the ability to detect nonlinear associations, we defined the smoothing parameter for each thin plate smoothing spline by conducting generalized cross validation. Each outcome was fit in a separate model. To test for independent associations between school grades and outcomes, we fit multivariate models: for mean grade point average, we included the mean grade point average and all covariates; and for specific grades, we included all specific grades and all covariates. Effect sizes were quantified as risk ratios (RR) with confidence intervals (CI) by contrasting each z‐score from the spline function to a z‐score of 0. As there were 21 tests (the mean grade point average and six specific subjects tested for three outcomes; 7 x 3) and we focused on confidence intervals (CI) instead of p‐values, we applied Bonferroni‐correction to the nominal 0.05 alpha level of the confidence intervals (CI), equal to alpha = 0.002 or 99.98% CIs (0.05/21 = 0.00238). For descriptive purposes, we also show conducting analyses with noncorrected CIs.

We conducted all analyses with R (version 3.6.3) including package *mgcv* for implementing GAMs with thin plate smoothing splines. Code to reproduce all analyses with a simulated data is available online at github.com/davgyl/school_sim (DOI: 10.5281/zenodo.6947470).

## 3 RESULTS

### 3.1 Participants

**FIGURE 1:**
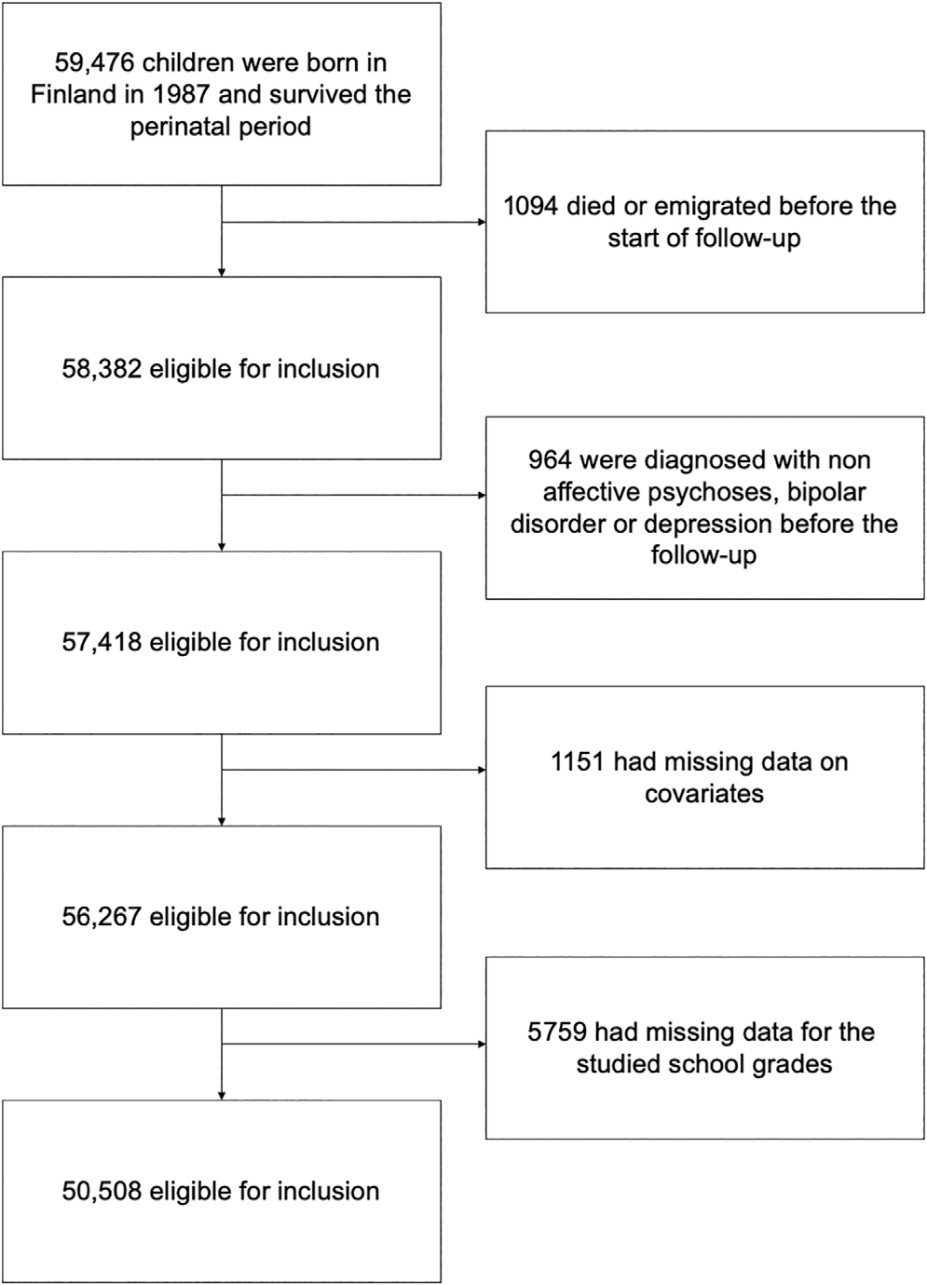
Flow chart of the exclusion criteria and the included participants in the cohort

In Finland in 1987, 59,476 children were born and survived the perinatal period. The exclusion of subjects is shown in Figure 1. After applying exclusion criteria, 50,508 cohort members were included in the analyses. In total, 727 individuals were diagnosed with psychosis, 489 with bipolar disorder and 3492 with depression during the follow‐up. The most common comorbid conditions were depression among those diagnosed with nonaffective psychoses (43.8%, n = 319/727) and depression among those diagnosed with bipolar disorder (51.3%, n = 251/489) (Table S1). The person‐years are described in Table S2.

### 3.2 Descriptive Results by Covariate Status

Table 1 describes the standardized mean grade point average and the cumulative incidence rates of outcomes among cohort members that were included in the analysis. The mean grade point average was lower for those who were male and lived in rural areas and those whose parents had low education level or history of nonaffective psychosis, bipolar disorder or depression. As further described in Table 1, the cumulative incidence of diagnosed nonaffective psychoses, bipolar disorder and depression was 1.46%, 0.99% and 7.02%, respectively, at the end of follow‐up in the studied sample. The cumulative incidence of diagnosed nonaffective psychoses was higher among males than females (1.62% vs. 1.30%), while the opposite pattern was seen for the other diagnoses (1.34% among females vs. 0.64% among males for bipolar disorder; 9.25% vs. 4.83% for depression; Table 1). All three disorders were more frequently diagnosed among those with parental history of psychiatric disorders, low parental education level and living in an urban area (Table 1). The mean (SD) ages of the first diagnosis of nonaffective psychoses, bipolar disorder and depression were 22.59 (3.33), 23.27 (2.93) and 22.11 (3.56) years, respectively.

**TABLE 1:** Description of the cohort members included in the analysis (*n* = 50,508) by covariate status

	Total	Standardized mean grade point average	Nonaffective psychosis	Bipolar disorder	Depression
Characteristic	No.	Mean (SD)	No. (cumulative incidence %)	No. (cumulative incidence %)	No. (cumulative incidence %)
Total sample	50,508	0.00 (1.00)	727 (1.46)	489 (0.99)	3492 (7.02)
Sex					
Females	25,052	0.31 (0.93)	320 (1.30)	328 (1.34)	2280 (9.25)
Males	25,456	−0.31 (0.97)	407 (1.62)	161 (0.64)	1212 (4.83)
Urbanicity of residence					
Rural	19,643	−0.08 (0.99)	231 (1.19)	166 (0.86)	1218 (6.28)
Urban	30,865	0.05 (1.00)	496 (1.63)	323 (1.07)	2274 (7.48)
Other psychiatric diagnosis before follow‐up	1960	−0.45 (1.06)	77 (4.00)	54 (2.82)	342 (17.7)
Parental education					
Primary	3420	−0.54 (0.96)	55 (1.63)	44 (1.31)	312 (9.24)
Secondary	21,429	−0.28 (0.97)	294 (1.39)	222 (1.05)	1564 (7.38)
Tertiary	25,659	0.30 (0.93)	378 (1.50)	223 (0.89)	1616 (6.41)
Parental psychiatric diagnosis before follow‐up	7603	−0.21 (1.03)	190 (2.54)	128 (1.72)	850 (11.36)

*Notes*:The cumulative incidence of diagnosed nonaffective psychosis, bipolar disorder and depression refers to at the end of follow‐up. The cumulative incidence at the end of follow‐up was calculated with Kaplan–Meier technique.

*Abbreviation*: SD, standard deviation.

### 3.3 Descriptive Results of School Grades

**FIGURE 2:**
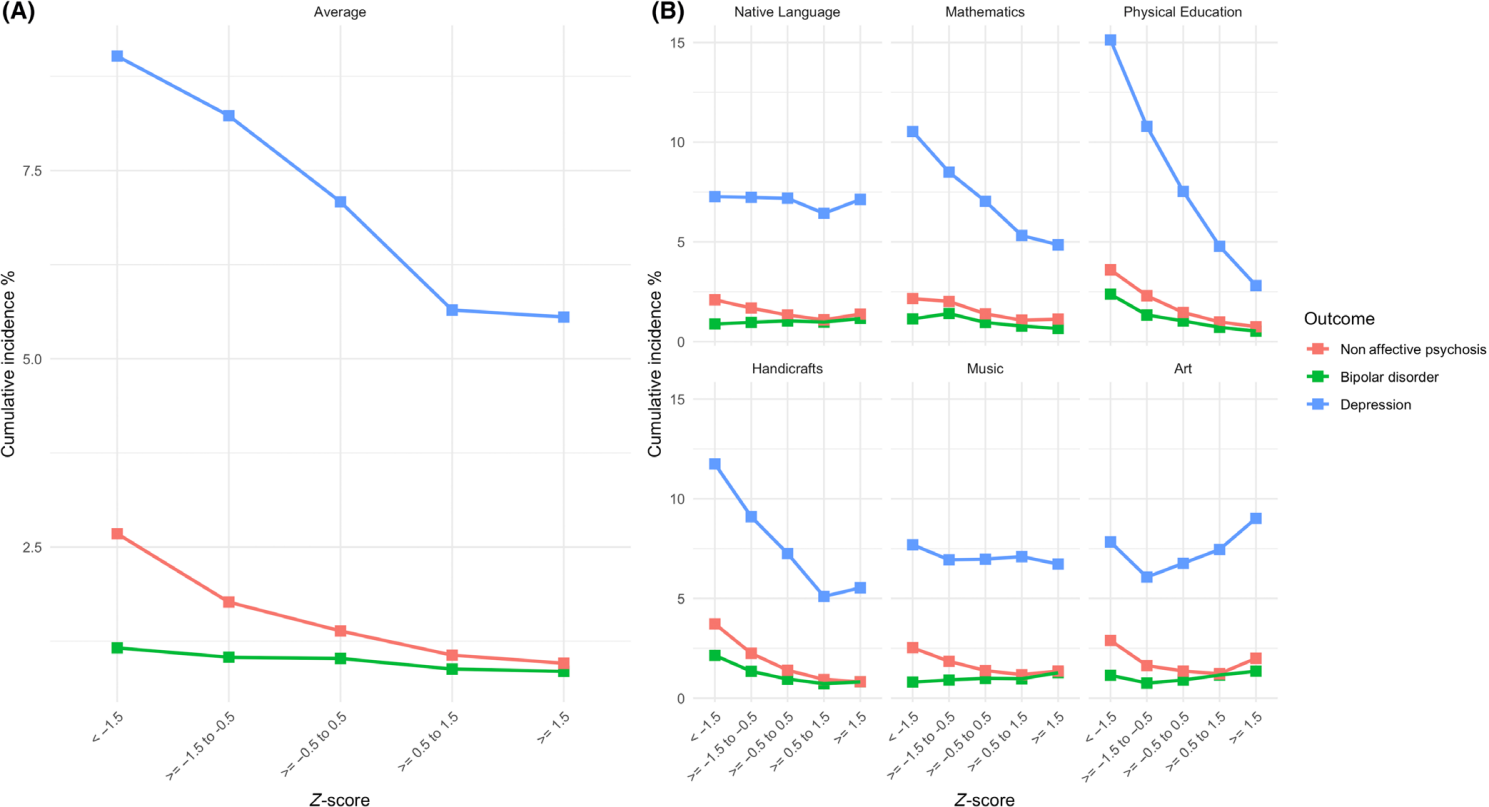
The cumulative incidence of diagnosed nonaffective psychosis, bipolar disorder and depression at the end of follow‐up when stratified by groups of z‐scores at <−1.5, −1.5 to <−0.5, −0.5 to <0.5, 0.5 to <1.5 and ≥1.5 of average (A) and specific (B) school subjects at age 15–16 years

The mean (SD) of the mean grade point average for those with diagnosed nonaffective psychoses, bipolar disorder and depression were − 0.31 (1.05), −0.11 (1.00) and − 0.18 (1.00), respectively. The cumulative incidence of the studied diagnoses stratified by groups of standardized school grades is shown in Table 2 and Figure 2. Among those with the lowest vs. highest mean grade point averages (−1.5 SD below vs. 1.5 SD over the mean, that is, <−1.5 z‐scores and ≥1.5 z‐scores), the cumulative incidences of diagnosed nonaffective psychoses were 2.68% vs. 0.96%, while the corresponding figures were 1.16% vs. 0.85% for bipolar disorder and 9.02% vs. 5.55% for depression. The highest cumulative incidences for all studied outcomes were seen for those with −1.5 SD below the mean of Physical Education grades (3.60% for nonaffective psychosis; 2.37% for bipolar disorder; and 15.13% for depression). Further description of the school grades can be found in the supplement (Figure S1 and Table S3).

**TABLE 2:** The number of subjects and the cumulative incidence of diagnosed nonaffective psychosis, bipolar disorder and depression at the end of follow‐up by pooled groups of school grades at age 15–16 years

		Nonaffective psychosis	Bipolar disorder	Depression
*Z*‐score of grade	Total no.	No. (cumulative incidence %)	No. (cumulative incidence %)	No. (cumulative incidence %)
Mean grade point average				
<−1.5	3934	104 (2.68)	45 (1.16)	351 (9.02)
−1.5 to −0.5	11,825	207 (1.77)	121 (1.04)	964 (8.23)
−0.5 to 0.5	17,640	241 (1.39)	177 (1.02)	1234 (7.08)
0.5 to 1.5	14,223	148 (1.06)	122 (0.88)	787 (5.65)
≥1.5	2886	27 (0.96)	24 (0.85)	156 (5.55)
Native language				
<−1.5	7269	150 (2.09)	63 (0.88)	522 (7.27)
−1.5 to −0.5	11,798	196 (1.68)	112 (0.96)	845 (7.23)
−0.5 to 0.5	16,210	212 (1.33)	165 (1.04)	1150 (7.19)
0.5 to 1.5	12,843	137 (1.09)	122 (0.97)	809 (6.43)
≥1.5	2388	32 (1.37)	27 (1.16)	166 (7.13)
Mathematics				
<−1.5	3663	78 (2.15)	41 (1.13)	382 (10.53)
−1.5 to −0.5	8588	171 (2.01)	119 (1.41)	722 (8.50)
−0.5 to 0.5	23,647	323 (1.39)	222 (0.96)	1641 (7.04)
0.5 to 1.5	11,335	119 (1.07)	86 (0.78)	592 (5.32)
≥1.5	3275	36 (1.12)	21 (0.65)	155 (4.85)
Physical education				
<−1.5	2311	82 (3.60)	54 (2.37)	346 (15.13)
−1.5 to −0.5	7937	180 (2.30)	104 (1.33)	847 (10.79)
−0.5 to 0.5	18,911	272 (1.46)	192 (1.03)	1405 (7.53)
0.5 to 1.5	15,727	152 (0.99)	110 (0.71)	739 (4.78)
≥1.5	5622	41 (0.75)	29 (0.53)	155 (2.81)
Handicrafts				
<−1.5	1473	54 (3.73)	31 (2.14)	171 (11.75)
−1.5 to −0.5	9154	203 (2.25)	121 (1.34)	822 (9.10)
−0.5 to 0.5	22,994	317 (1.40)	215 (0.95)	1646 (7.26)
0.5 to 1.5	15,259	140 (0.93)	109 (0.73)	765 (5.10)
≥1.5	1628	13 (0.82)	13 (0.82)	88 (5.53)
Art				
<−1.5	1689	48 (2.89)	19 (1.15)	130 (7.83)
−1.5 to −0.5	9557	154 (1.63)	71 (0.75)	573 (6.07)
−0.5 to 0.5	21,406	286 (1.35)	191 (0.91)	1431 (6.77)
0.5 to 1.5	14,510	174 (1.22)	164 (1.16)	1063 (7.46)
≥1.5	3346	65 (2.00)	44 (1.35)	295 (9.02)
Music				
<−1.5	2408	60 (2.53)	19 (0.80)	183 (7.69)
−1.5 to −0.5	9716	177 (1.84)	87 (0.91)	667 (6.94)
−0.5 to 0.5	18,916	258 (1.38)	185 (0.99)	1302 (6.97)
0.5 to 1.5	15,026	173 (1.18)	143 (0.97)	1047 (7.10)
≥1.5	4442	59 (1.36)	55 (1.27)	293 (6.73)

*Notes*: The cumulative incidence at the end of follow‐up was calculated with Kaplan–Meier technique.

### 3.4 Independent Associations between School Grades and Later Diagnoses

**FIGURE 3:**
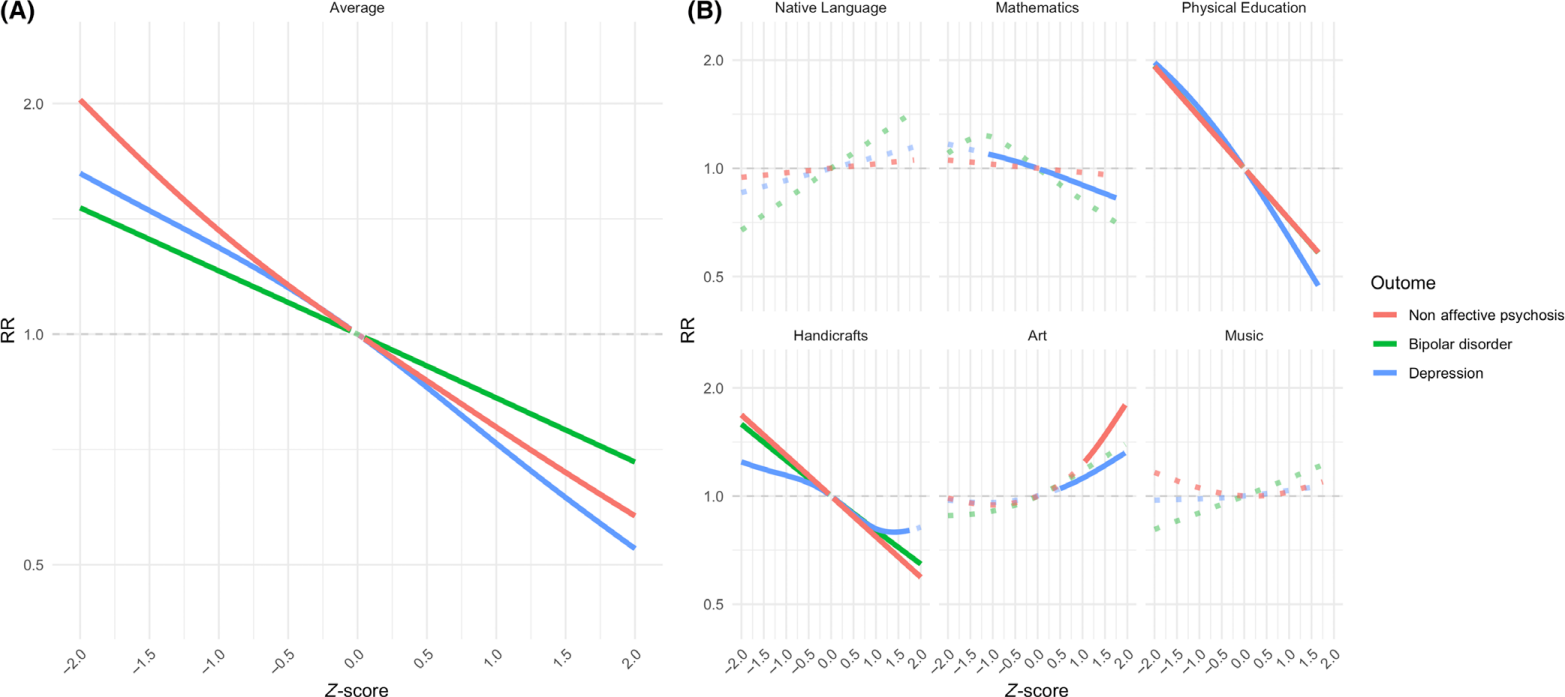
Independent associations for mean grade point average (A) and specific school grades (B) in relation to diagnosed nonaffective psychoses, bipolar disorder and depression. The solid line denotes estimates that have Bonferroni‐corrected confidence (CI) intervals not including 1, and the dotted line denotes estimates with corresponding CIs crossing 1 (CIs not shown). The school grades were modeled with smoothing splines in general additive models. In the multivariate models, the covariates included sex, level of urbanicity, parental education level, any psychiatric diagnosis before start of follow‐up among participants and parental history of any psychiatric diagnosis. In the multivariate model shown in panel (A), the predictors included mean grade point average and the covariates. In the multivariate model shown in panel (B), the predictors included specific school grades and the covariates. Abbreviations: RR, risk ratio

The multivariate models examining independent associations between mean grade point average and outcomes included the following covariates: sex, level of urbanicity, parental education level, any psychiatric diagnosis before start of follow‐up among participants and parental history of any psychiatric diagnosis. As summarized in Figure 3A, there was an inverse linear association between MPGA and all outcomes (effect sizes as numbers are shown in Table S4). When comparing to MPGA at 0 in these models, the MPGA at 1.5 were associated with later nonaffective psychoses (RR = 1.65; Bonferroni‐corrected CI, 1.31–2.07), bipolar disorder (1.33; 1.06–1.67) and depression (1.45; 1.28–1.64) (Figure 3A).

In the models assessing independent associations between specific school subjects and later outcomes, the following covariates were included: all school subjects and sex, level of urbanicity, parental education level, any psychiatric diagnosis before start of follow‐up among participants and parental history of any psychiatric diagnosis. While low grades in the academic subject Mathematics were overrepresented among those diagnosed with nonaffective psychoses, bipolar disorder and depression in the descriptive analyses (Figure 2 and Table 2), the associations were only significant for depression in multivariate analyses (Figure 3B). However, Physical Education were negatively associated with all studied outcomes in the multivariate model and showed the highest RRs (1.63, Bonferroni‐corrected CI 1.36–1.95, for nonaffective psychosis; 1.64, 1.30–2.05, for bipolar disorder; 1.72, 1.53–1.93, for depression) (Figure 3B). Interestingly, grades in Art were nonlinearly associated with nonaffective psychoses and depression as high grades 1.5 SD over the mean showed an association (RR = 1.48; Bonferroni‐corrected CI, 1.11–1.96 for nonaffective psychosis; RR = 1.22; Bonferroni‐corrected CI, 1.07–1.38 for depression) (Figure 3B).

### 3.5 Additional analyses

To test whether the findings were similar for males and females, we stratified analyses by sex; the findings were in line with the main results (Figures S2 and S3). To examine whether Physical Education was driving the associations between MPGA and outcomes, we analyzed associations for a mean grade point average variable of the five specific school subjects excluding Physical Education, but the findings were similar to the main results (Figure S4). Finally, for a more exploratory approach, we also present the main associations when no Bonferroni‐correction has been made to the CIs (Figure S5).

## 4 DISCUSSION

In a total population study of all individuals born in Finland in 1987, we found that mean grade point average was associated with later diagnoses of psychosis, bipolar disorder and depression in a linear way: the lower the grade was, the higher the predictive value was for all studied outcomes. When assessing specific school subjects, the largest independent effect sizes were detected for Physical Education, not academic school subjects, with poorer scores predicting all disorder outcomes. Interestingly, grades in Art were also nonlinearly associated with diagnosed nonaffective psychoses and depression but, in this case, with higher grades predicting diagnoses. These findings demonstrate that school performance is associated with all three studied major diagnoses and show that specific types of school performance have both linear and nonlinear associations with later mental disorders.

Grades in Physical Education were inversely associated with all studied outcomes. Similarly, grades in Handicrafts were inversely associated with later diagnoses of psychosis and bipolar disorder. There are a number of reasons why poorer performance in Physical Education and Handicrafts might predict later mental disorders. Subtle neuromotor deficits may be reflected in performance in both Physical Education and Handicrafts, and delays or deficits in neuromotor development are robust predictors for psychotic disorders.^7^ While the effects were strongest for nonaffective psychoses, they were also present for bipolar disorder and depression, even though the latter are not typically preceded by deficits in neuromotor development based on motor tests.^24^, ^25^, ^26^ At the same time, motor development disorders have been associated with bipolar disorder and depression.^27^, ^28^ It might also be the case that deficits in motivation explain poorer performance in some subjects more than others, such as Physical Education.^29^

The current study adds to the literature by showing that high grades in Art independently predict diagnoses of psychosis and depression at age 15–16 years. This is in line with a Swedish study showing that studying a creative subject at high school or university is associated with later diagnoses of psychosis, bipolar disorder and depression.^10^ One possible explanation for the association between creativity and psychiatric diagnoses is that both might have overlapping genetic causes. For example, one study has shown that a family history of psychiatric disorders is associated with creativity,^30^ while another has shown that people with high polygenic risk scores for schizophrenia and bipolar disorder more often work in a creative occupation.^31^ Another possible psychological explanation that has been presented for the association between creativity and psychosis is that some cognitive styles, such as making original linking between different ideas, may be related to both creative thinking and psychosis.^10^

Strengths of the study include the nationwide cohort, the approach to study multiple major psychiatric disorders, and the ability to study both linear and nonlinear associations. The following limitations should be considered. First, while we were able to study several specific school grades reflecting different types of abilities, we did not have direct measures of cognitive performance from neuropsychological tests. Second, a tenth of the cohort had missing school grades at start of follow‐up including those who did not complete the spring they turned 16 years. These cohort members are likely to have low school performance, because a common reason for not having complete school grades is extending primary to more than to the usual 9 years. Therefore, the associations between low grades and the studied outcomes might be underestimated. Third, the studied disorders were diagnosed in specialized services. Most psychosis and bipolar diagnoses occur in specialized services,^32^ but this is not necessarily the case for depression, which is often diagnosed and treated in primary care, that is, in clinics that are not included in the register. Fourth, we followed the sample to age 28 years; future studies with longer follow‐up should assess whether the findings are generalizable to disorders diagnosed at an older age. Fifth, we used a full birth cohort born in 1 year with comprehensive information from several registers, but even then our sample size was not sufficient to study more specific disorders such as schizophrenia and bipolar type I. Future studies with larger sample size should assess whether the results generalize to these disorders. However, it is noteworthy that we found similar patterns across all studied diagnoses.

The study shows that specific types of school performance predict diagnoses of psychosis, bipolar disorder and depression in a rather similar fashion with little variation. Grades in Physical Education showed an inverse linear association with all studied diagnoses and high grades in Art predicted psychosis and depression. This indicates that nonlinear associations of specific types of school performance should be considered sources of information when mapping precursors for several severe psychiatric disorders.

## CONFLICT OF INTEREST

The authors report no biomedical financial interests or potential conflicts of interest.

### PEER REVIEW

The peer review history for this article is available at https://publons.com/publon/10.1111/acps.13481.

## Supporting information


**Appendix S1** Supporting Information

## Data Availability

Individual‐level register‐based data cannot be freely shared because of Finnish legislation. Code to produce a simulated data is available online at github.com/davgyl/school_sim (DOI: 10.5281/zenodo.6947470).

## References

[1] MacCabe JH , Lambe MP , Cnattingius S , et al. Scholastic achievement at age 16 and risk of schizophrenia and other psychoses: a national cohort study. Psychol Med. 2008;38(8):1133‐1140. doi:10.1017/S0033291707002048 17988422

[2] MacCabe JH , Lambe MP , Cnattingius S , et al. Excellent school performance at age 16 and risk of adult bipolar disorder: national cohort study. Br J Psychiatry. 2010;196(2):109‐115. doi:10.1192/bjp.bp.108.060368 20118454

[3] Kendler KS , Ohlsson H , Mezuk B , Sundquist K , Sundquist J . A Swedish National Prospective and co‐relative study of school achievement at age 16, and risk for schizophrenia, other nonaffective psychosis, and bipolar illness. Schizophr Bull. 2016;42(1):77‐86. doi:10.1093/schbul/sbv103 26231719 PMC4681557

[4] Cannon M , Jones P , Huttunen MO , et al. School performance in Finnish children and later development of schizophrenia: a population‐based longitudinal study. Arch Gen Psychiatry. 1999;56(5):457‐463. doi:10.1001/archpsyc.56.5.457 10232301

[5] Sorberg Wallin A , Koupil I , Gustafsson J‐E , Zammit S , Allebeck P , Falkstedt D . Academic performance, externalizing disorders and depression: 26,000 adolescents followed into adulthood. Soc Psychiatry Psychiatr Epidemiol. 2019;54(8):977‐986. doi:10.1007/s00127-019-01668-z 30783692

[6] Zammit S , Allebeck P , David AS , et al. A longitudinal study of premorbid IQ score and risk of developing schizophrenia, bipolar disorder, severe depression, and other nonaffective psychoses. Arch Gen Psychiatry. 2004;61(4):354‐360. doi:10.1001/archpsyc.61.4.354 15066893

[7] Dickson H , Laurens KR , Cullen AE , Hodgins S . Meta‐analyses of cognitive and motor function in youth aged 16 years and younger who subsequently develop schizophrenia. Psychol Med. 2012;42(4):743‐755. doi:10.1017/S0033291711001693 21896236

[8] Filatova S , Koivumaa‐Honkanen H , Hirvonen N , et al. Early motor developmental milestones and schizophrenia: a systematic review and meta‐analysis. Schizophr Res. 2017;188:13‐20. doi:10.1016/j.schres.2017.01.029 28131598

[9] Lassila M , Nordström T , Hurtig T , et al. School success in childhood and subsequent prodromal symptoms and psychoses in the northern Finland birth cohort 1986. Psychol Med. 2020;50(6):948‐955. doi:10.1017/S0033291719000825 31010450

[10] MacCabe JH , Sariaslan A , Almqvist C , Lichtenstein P , Larsson H , Kyaga S . Artistic creativity and risk for schizophrenia, bipolar disorder and unipolar depression: a Swedish population‐based case‐control study and sib‐pair analysis. Br J Psychiatry. 2018;212(6):370‐376. doi:10.1192/bjp.2018.23 29697041

[11] Gyllenberg D , Marttila M , Sund R , et al. Temporal changes in the incidence of treated psychiatric and neurodevelopmental disorders during adolescence: an analysis of two national Finnish birth cohorts. Lancet Psychiatry. 2018;5(3):227‐236. doi:10.1016/S2215-0366(18)30038-5 29398636

[12] Sund R . Quality of the Finnish hospital discharge register: a systematic review. Scand J Public Health. 2012;40(6):505‐515. doi:10.1177/1403494812456637 22899561

[13] Mäkikyro T , Isohanni M , Moring J , Hakko H , Hovatta I , Lönnqvist J . Accuracy of register‐based schizophrenia diagnoses in a genetic study. Eur Psychiatry. 1998;13(2):57‐62. doi:10.1016/S0924-9338(98)80019-9 19698600

[14] Kieseppä T , Partonen T , Kaprio J , Lönnqvist J . Accuracy of register‐ and record‐based bipolar I disorder diagnoses in Finland; a study of twins. Acta Neuropsychiatr. 2000;12(3):106‐109. doi:10.1017/S0924270800035535 26975265

[15] Järvinen, R. *Current trends in inclusive educaton in Finland*. Paper presented at the Finland: Regional Preparatory Workshop on Inclusive Educa6on. Eastern and South Eastern Europe, Sinaia, Romania, 14–16 June 2007. Available http://www.ibe.unesco.org/fileadmin/user_upload/Inclusive_Education/Reports/sinaia_07/finland_inclusion_07.pdf. Accessed March 30, 2022, DOI: 10.3389/fpubh.2022.858210

[16] McGrath JJ , Wray NR , Pedersen CB , Mortensen PB , Greve AN , Petersen L . The association between family history of mental disorders and general cognitive ability. Transl Psychiatry. 2014;4:e412. doi:10.1038/tp.2014.60 25050992 PMC4119227

[17] McGrath J , Saha S , Welham J , El Saadi O , MacCauley C , Chant D . A systematic review of the incidence of schizophrenia: the distribution of rates and the influence of sex, urbanicity, migrant status and methodology. BMC Med. 2004;2:13. 15115547 10.1186/1741-7015-2-13 PMC421751

[18] Eaton W , Harrison G . Life chances, life planning, and schizophrenia. International Journal of Mental Health. 2001;30(1):58‐81.

[19] Heinz A , Deserno L , Reininghaus U . Urbanicity, social adversity and psychosis. World Psychiatry. 2013;12(3):187‐197. doi:10.1002/wps.20056 24096775 PMC3799240

[20] Dalsgaard S , Thorsteinsson E , Trabjerg BB , et al. Incidence rates and cumulative incidences of the full Spectrum of diagnosed mental disorders in childhood and adolescence. JAMA Psychiat. 2020;77(2):155‐164. doi:10.1001/jamapsychiatry.2019.3523 PMC6902162 31746968

[21] Dalsgaard S , McGrath J , Ostergaard SD , et al. Association of Mental Disorder in childhood and adolescence with subsequent educational achievement. JAMA Psychiat. 2020;77(8):797‐805. doi:10.1001/jamapsychiatry.2020.0217 PMC7097843 32211833

[22] Finland's Environmental Administration. Urban‐rural classification. 2014; https://www.ymparisto.fi/en-US/Living_environment_and_planning/Community_structure/Information_about_the_community_structure/Urbanrural_classification. Accessed June 1, 2020.

[23] Wood SN . Thin plate regression splines. J R Stat Soc Series B Stat Methodology. 2003;65(1):95‐114.

[24] Murray RM , Sham P , Van Os J , Zanelli J , Cannon M , McDonald C . A developmental model for similarities and dissimilarities between schizophrenia and bipolar disorder. Schizophr Res. 2004;71(2–3):405‐416. 15474912 10.1016/j.schres.2004.03.002

[25] Cannon M , Caspi A , Moffitt TE , et al. Evidence for early‐childhood, pan‐developmental impairment specific to schizophreniform disorder: results from a longitudinal birth cohort. Arch Gen Psychiatry. 2002;59(5):449‐456. doi:10.1001/archpsyc.59.5.449 11982449

[26] Meier MH , Caspi A , Reichenberg A , et al. Neuropsychological decline in schizophrenia from the premorbid to the postonset period: evidence from a population‐representative longitudinal study. Am J Psychiatry. 2014;171(1):91‐101. doi:10.1176/appi.ajp.2013.12111438 24030246 PMC3947263

[27] Gundel LK , Pedersen CB , Munk‐Olsen T , Dalsgaard S . Longitudinal association between mental disorders in childhood and subsequent depression ‐ a nationwide prospective cohort study. J Affect Disord. 2018;227:56‐64. doi:10.1016/j.jad.2017.10.023 29053976

[28] Maibing CF , Pedersen CB , Benros ME , Mortensen PB , Dalsgaard S , Nordentoft M . Risk of schizophrenia increases after all child and adolescent psychiatric disorders: a Nationwide study. Schizophr Bull. 2015;41(4):963‐970. doi:10.1093/schbul/sbu119 25193974 PMC4466169

[29] Ntoumanis N . A self‐determination approach to the understanding of motivation in physical education. Br J Educ Psychol. 2001;71(Pt 2):225‐242. doi:10.1348/000709901158497 11449934

[30] Kyaga S , Lichtenstein P , Boman M , Hultman C , Langstrom N , Landen M . Creativity and mental disorder: family study of 300,000 people with severe mental disorder. Br J Psychiatry. 2011;199(5):373‐379. doi:10.1192/bjp.bp.110.085316 21653945

[31] Power RA , Steinberg S , Bjornsdottir G , et al. Polygenic risk scores for schizophrenia and bipolar disorder predict creativity. Nat Neurosci. 2015;18(7):953‐955. doi:10.1038/nn.4040 26053403

[32] Perälä J , Suvisaari J , Saarni SI , et al. Lifetime prevalence of psychotic and bipolar I disorders in a general population. Arch Gen Psychiatry. 2007;64(1):19‐28. doi:10.1001/archpsyc.64.1.19 17199051

