# Supplementary material for: School performance and later diagnoses of nonaffective psychoses, bipolar disorder, and depression
Source: Acta Psychiatr Scand. 2022 Aug 7;146(5):420–9. doi: 10.1111/acps.13481 (PMC9804878; doi:10.1111/acps.13481)
Supplement: Supplementary file 1 — Appendix S1 Supporting Information [file ACPS-146-420-s001.docx]

**SUPPLEMENTARY MATERIAL**

**School Performance and Later Diagnoses of Non-Affective Psychoses, Bipolar Disorder and Depression**

***Diagnostic codes***

The ICD codes for “any psychiatric diagnosis” were

- ICD-10: F00-F59, F60-F63, F68-F69, F80-F99
- ICD-9: 295-301, 303-316
- ICD-8: 295-301, 303-305, 30600, 30610, 30620, 30630, 30640, 30650, 30660, 30670, 307-309

| **Supplemental Table 1.** Comorbidity between the outcomes. | | | | |
| --- | --- | --- | --- | --- |
| Combination | No. | % of non-affective psychosis (n=727) | % of bipolar disorder (n=489) | % of depression (n=3,492) |
| Non-affective psychosis, bipolar disorder and depression | 76 | 10.5 | 15.5 | 2.2 |
| Non-affective psychosis and bipolar disorder | 42 | 5.8 | 8.6 | – |
| Non-affective psychosis and depression | 319 | 43.9 | – | 9.1 |
| Bipolar disorder and depression | 251 | – | 51.3 | 7.2 |
| Non-affective psychosis only | 290 | 39.9 | – | – |
| Bipolar disorder only | 120 | – | 24.5 | – |
| Depression only | 2846 | – | – | 81.5 |
| Total | 3944 | 100.0 | 100.0 | 100.0 |

| **Supplemental Table 2.** The person-years used in the time-to-event analyses of diagnosed non-affective psychosis, bipolar disorder and depression. | | | | |
| --- | --- | --- | --- | --- |
|  |  | Non-affective psychosis | Bipolar disorder | Depression |
| Characteristic | Total No. | Person-years | Person-years | Person-years |
| Total sample | 50,508 | 621,925.76 | 623,590.50 | 604,276.60 |
| Sex |  |  |  |  |
| Females | 25,052 | 307,657.62 | 307,959.25 | 294,533.93 |
| Males | 25,456 | 314,268.14 | 315,631.24 | 309,742.67 |
| Urbanicity of residence |  |  |  |  |
| Rural | 19,643 | 242,415.74 | 242,915.18 | 236,137.22 |
| Urban | 30,865 | 379,510.02 | 380,675.32 | 368,139.38 |
| Other psychiatric diagnosis before follow-up | 1,960 | 23,756.18 | 23,971.84 | 21,853.39 |
| Parental education |  |  |  |  |
| Primary | 3,420 | 42,127.61 | 42,219.80 | 40,403.41 |
| Secondary | 21,429 | 264,851.41 | 265,393.74 | 256,690.54 |
| Tertiary | 25,659 | 314,946.74 | 315,976.96 | 307,182.65 |
| Parental psychiatric before follow-up | 7,603 | 93,011.02 | 93,452.23 | 88,548.94 |

| **Supplemental Table 2** continued. | | | | |
| --- | --- | --- | --- | --- |
|  |  | Non-affective psychosis | Bipolar disorder | Depression |
| Characteristic | Total No. | Person-years | Person-years | Person-years |
| Mean grade point average (z-score) |  |  |  |  |
| <-1.5 | 3,934 | 48,448.48 | 48,744.06 | 46,836.60 |
| -1.5 – -0.5 | 11,825 | 146,132.49 | 146,706.49 | 141,301.46 |
| -0.5 – 0.5 | 17,640 | 217,582.24 | 218,113.43 | 211,172.95 |
| 0.5 – 1.5 | 14,223 | 174,495.28 | 174,736.92 | 170,456.56 |
| ≥1.5 | 2,886 | 35,267.27 | 35,289.59 | 34,509.04 |
| Native Language (z-score) |  |  |  |  |
| <-1.5 | 7,269 | 89,615.39 | 90,074.02 | 87,284.74 |
| -1.5 – -0.5 | 11,798 | 145,707.95 | 146,262.87 | 141,502.21 |
| -0.5 – 0.5 | 16,210 | 199,988.69 | 200,396.85 | 194,003.09 |
| 0.5 – 1.5 | 12,843 | 157,510.14 | 157,708.65 | 153,222.52 |
| ≥1.5 | 2,388 | 29,103.59 | 29,148.09 | 28,264.05 |
| Mathematics (z-score) |  |  |  |  |
| <-1.5 | 3,663 | 45,231.74 | 45,450.33 | 43,317.41 |
| -1.5 – -0.5 | 8,588 | 105,921.74 | 106,290.77 | 102,379.80 |
| -0.5 – 0.5 | 23,647 | 291,264.26 | 291,997.41 | 282,749.09 |
| 0.5 – 1.5 | 11,335 | 139,341.38 | 139,597.00 | 136,335.37 |
| ≥1.5 | 3,275 | 40,166.65 | 40,254.98 | 39,494.93 |

| **Supplemental Table 2** continued. | | | | |
| --- | --- | --- | --- | --- |
|  |  | Non-affective psychosis | Bipolar disorder | Depression |
| Characteristic | Total No. | Person-years | Person-years | Person-years |
| Physical Education (z-score) | |  |  |  |
| <-1.5 | 2,311 | 28,264.68 | 28,440.75 | 26,342.58 |
| -1.5 – -0.5 | 7,937 | 97,593.34 | 98,167.93 | 93,479.93 |
| -0.5 – 0.5 | 18,911 | 232,909.31 | 233,543.54 | 225,578.21 |
| 0.5 – 1.5 | 15,727 | 193,868.53 | 194,117.87 | 190,261.38 |
| ≥1.5 | 5,622 | 69,289.90 | 69,320.41 | 68,614.50 |
| Handicrafts (z-score) |  |  |  |  |
| <-1.5 | 1,473 | 17,974.03 | 18,095.76 | 17,089.83 |
| -1.5 – -0.5 | 9,154 | 112,453.95 | 113,025.52 | 108,506.90 |
| -0.5 – 0.5 | 22,994 | 283,358.02 | 284,089.22 | 274,893.05 |
| 0.5 – 1.5 | 15,259 | 188,210.56 | 188,448.01 | 184,357.60 |
| ≥1.5 | 1,628 | 19,929.20 | 19,931.99 | 19,429.22 |
| Art (z-score) |  |  |  |  |
| <-1.5 | 1,689 | 20,659.25 | 20,795.92 | 20,166.01 |
| -1.5 – -0.5 | 9,557 | 117,961.32 | 118,486.52 | 115,439.26 |
| -0.5 – 0.5 | 21,406 | 264,288.52 | 264,943.73 | 256,820.10 |
| 0.5 – 1.5 | 14,510 | 178,267.19 | 178,477.80 | 172,582.92 |
| ≥1.5 | 3,346 | 40,749.47 | 40,886.52 | 39,268.32 |
| Music (z-score) |  |  |  |  |
| <-1.5 | 2,408 | 29,639.68 | 29,849.75 | 28,884.45 |
| -1.5 – -0.5 | 9,716 | 119,857.27 | 120,403.92 | 116,671.03 |
| -0.5 – 0.5 | 18,916 | 233,511.85 | 234,116.39 | 227,077.75 |
| 0.5 – 1.5 | 15,026 | 184,498.68 | 184,729.66 | 178,752.54 |
| ≥1.5 | 4,442 | 54,418.28 | 54,490.78 | 52,890.83 |

**1**

| **Supplemental Table 3**. The original grades on scale from four (failed) to ten (excellent) and their corresponding z-scores for average scores (shown for integer values) and for each specific subject. | | | | | | | |
| --- | --- | --- | --- | --- | --- | --- | --- |
|  | Original grade and corresponding z-score | | | | | | |
| School subject | 4 | 5 | 6 | 7 | 8 | 9 | 10 |
| Mean grade point average | – | -3.14 | -2.04 | -0.94 | 0.16 | 1.26 | 2.37 |
| Native Language | -3.25 | -2.40 | -1.54 | -0.68 | 0.17 | 1.03 | 1.89 |
| Mathematics | -2.62 | -1.89 | -1.15 | -0.42 | 0.31 | 1.04 | 1.77 |
| Physical Education | -4.11 | -3.15 | -2.19 | -1.23 | -0.26 | 0.70 | 1.66 |
| Handicrafts | -4.83 | -3.66 | -2.49 | -1.32 | -0.15 | 1.03 | 2.20 |
| Art | -4.49 | -3.41 | -2.33 | -1.25 | -0.17 | 0.90 | 1.98 |
| Music | -4.13 | -3.14 | -2.15 | -1.17 | -0.18 | 0.81 | 1.80 |

**Supplemental Figure 1**. Pearson correlation between standardized mean grade point average and specific school subjects in the total analytic sample (n=50,508).

**Supplemental Figure 2**. The same analyses as in Figure 3, but restricted to males.

**Supplemental Figure 3**. The same analyses as in Figure 3, but restricted to females.

**Supplemental Figure 4**. Associations between MPGA and outcomes as in Figure 3A, but with a mean grade point average variable of the five specific school subjects excluding Physical Education.

**Supplemental Figure 5**. The present the main associations as in Figure 3 but with no Bonferroni-correction applied to the confidence intervals. The solid line denotes estimates that have non-corrected confidence (CI) intervals not including 1, and the dotted line denotes estimates with corresponding CIs crossing 1 (CIs not shown).

| **Supplemental Table 4.** Risk ratios (RR) and Bonferroni-corrected confidence intervals (CI) contrasting to z-score 0 as shown in Figure 3 in the manuscript. The mean grade point averages were modelled with smoothing splines in general additive models and the covariates consisted of sex, level of urbanicity, parental education level, psychiatric disorder before follow-up and parental any psychiatric diagnosis. The covariates in the model of specific school grades were the same as for mean grade point average and all school grades. Bold face and asterisk indicates statistically significant contrast with the Bonferroni-corrected CI not crossing 1. | | | | | |
| --- | --- | --- | --- | --- | --- |
|  | Non-affective psychosis |  | Bipolar disorder |  | Depression |
| School subject and z-score | RR (Bonferroni-corrected CI) |  | RR (Bonferroni-corrected CI) |  | RR (Bonferroni-corrected CI) |
| Mean grade point average |  |  |  |  |  |
| -1.5 | 1.65 (1.31 - 2.07)* |  | 1.33 (1.06 - 1.67)* |  | 1.45 (1.28 - 1.64)* |
| -0.5 | 1.16 (1.07 - 1.26)* |  | 1.10 (1.02 - 1.19)* |  | 1.15 (1.10 - 1.21)* |
| 0 | 1 |  | 1 |  | 1 |
| 0.5 | 0.87 (0.79 - 0.95)* |  | 0.91 (0.84 - 0.98)* |  | 0.85 (0.81 - 0.89)* |
| 1.5 | 0.66 (0.48 - 0.90)* |  | 0.75 (0.59 - 0.95)* |  | 0.61 (0.52 - 0.71)* |
| Native Language |  |  |  |  |  |
| -1.5 | 0.96 (0.74 - 1.24) |  | 0.74 (0.54 - 1.03) |  | 0.89 (0.79 - 1.01) |
| -0.5 | 0.99 (0.90 - 1.08) |  | 0.91 (0.81 - 1.01) |  | 0.96 (0.92 - 1.00) |
| 0 | 1 |  | 1 |  | 1 |
| 0.5 | 1.01 (0.93 - 1.11) |  | 1.10 (0.99 - 1.23) |  | 1.04 (1.00 - 1.08) |
| 1.5 | 1.04 (0.80 - 1.36) |  | 1.35 (0.97 - 1.86) |  | 1.12 (0.98 - 1.27) |
| Mathematics |  |  |  |  |  |
| -1.5 | 1.04 (0.82 - 1.33) |  | 1.21 (0.80 - 1.81) |  | 1.13 (0.99 - 1.28) |
| -0.5 | 1.01 (0.93 - 1.10) |  | 1.12 (0.94 - 1.32) |  | 1.05 (1.00 - 1.09)* |
| 0 | 1 |  | 1 |  | 1 |
| 0.5 | 0.99 (0.91 - 1.07) |  | 0.90 (0.77 - 1.06) |  | 0.95 (0.91 - 0.99)* |
| 1.5 | 0.96 (0.75 - 1.22) |  | 0.74 (0.45 - 1.21) |  | 0.85 (0.74 - 0.98)* |

| **Supplemental Table 4** continued. | | | | | |
| --- | --- | --- | --- | --- | --- |
|  | Non-affective psychosis |  | Bipolar disorder |  | Depression |
| School subject and z-score | RR (Bonferroni-corrected CI) |  | RR (Bonferroni-corrected CI) |  | RR (Bonferroni-corrected CI) |

| Physical Education |  |  |  |  |  |
| --- | --- | --- | --- | --- | --- |
| -1.5 | 1.63 (1.36 - 1.95)* |  | 1.64 (1.30 - 2.05)* |  | 1.72 (1.53 - 1.93)* |
| -0.5 | 1.18 (1.11 - 1.25)* |  | 1.18 (1.09 - 1.27)* |  | 1.22 (1.17 - 1.28)* |
| 0 | 1 |  | 1 |  | 1 |
| 0.5 | 0.85 (0.80 - 0.90)* |  | 0.85 (0.79 - 0.92)* |  | 0.8 (0.76 - 0.85)* |
| 1.5 | 0.61 (0.51 - 0.73)* |  | 0.61 (0.49 - 0.77)* |  | 0.51 (0.43 - 0.60)* |
| Handicrafts |  |  |  |  |  |
| -1.5 | 1.48 (1.23 - 1.78)* |  | 1.41 (1.11 - 1.79)* |  | 1.19 (1.03 - 1.37)* |
| -0.5 | 1.14 (1.07 - 1.21)* |  | 1.12 (1.03 - 1.21)* |  | 1.09 (1.03 - 1.15)* |
| 0 | 1 |  | 1 |  | 1 |
| 0.5 | 0.88 (0.83 - 0.93)* |  | 0.89 (0.82 - 0.97)* |  | 0.89 (0.84 - 0.95)* |
| 1.5 | 0.68 (0.56 - 0.82)* |  | 0.72 (0.56 - 0.93)* |  | 0.79 (0.67 - 0.93)* |
| Art |  |  |  |  |  |
| -1.5 | 0.96 (0.72 - 1.28) |  | 0.89 (0.64 - 1.25) |  | 0.96 (0.84 - 1.11) |
| -0.5 | 0.96 (0.87 - 1.06) |  | 0.94 (0.85 - 1.05) |  | 0.97 (0.93 - 1.02) |
| 0 | 1 |  | 1 |  | 1 |
| 0.5 | 1.08 (0.96 - 1.21) |  | 1.08 (0.97 - 1.20) |  | 1.05 (1.00 - 1.10)* |
| 1.5 | 1.48 (1.11 - 1.96)* |  | 1.29 (0.95 - 1.74) |  | 1.22 (1.07 - 1.38)* |
| Music |  |  |  |  |  |
| -1.5 | 1.10 (0.86 - 1.41) |  | 0.85 (0.65 - 1.11) |  | 0.98 (0.87 - 1.09) |
| -0.5 | 1.02 (0.94 - 1.11) |  | 0.95 (0.87 - 1.03) |  | 0.99 (0.95 - 1.02) |
| 0 | 1 |  | 1 |  | 1 |
| 0.5 | 1.00 (0.92 - 1.10) |  | 1.06 (0.97 - 1.15) |  | 1.02 (0.98 - 1.05) |
| 1.5 | 1.07 (0.80 - 1.43) |  | 1.19 (0.91 - 1.54) |  | 1.06 (0.94 - 1.19) |
